# Infertility Treatments Resulting in Twin Pregnancy: Does It Increase the Risk for Future Childhood Malignancy †

**DOI:** 10.3390/jcm12113728

**Published:** 2023-05-29

**Authors:** Tal Shabtai, Eyal Sheiner, Tamar Wainstock, Arie Raziel, Roy Kessous

**Affiliations:** 1 Department of Obstetrics and Gynecology, Soroka University Medical Center, Ben-Gurion University of the Negev, POB 151, Beer-Sheva 84101, Israel; tshabtai@gmail.com (T.S.); sheiner@bgu.ac.il (E.S.); 2 The Department of Public Health, Faculty of Health Sciences, Ben-Gurion University of the Negev, Beer-Sheva 84105, Israel; wainstoc@bgu.ac.il; 3 IVF Unit, Wolfson Medical Center, Affiliated to Tel-Aviv University, Tel Aviv 69978, Israel; arier@wmc.gov.il

**Keywords:** reproductive technology, assisted pregnancy, twin, neoplasms, child

## Abstract

**Background:** Controversy exists in the literature regarding the possible association between infertility treatments in singleton pregnancies and long-term risk for childhood malignancy. Data regarding infertility treatments in twins and long-term childhood malignancies are scarce. **Objective:** We sought to investigate whether twins conceived following infertility treatments are at an increased risk for childhood malignancy. **Study design:** A population-based retrospective cohort study, comparing the risk for future childhood malignancy in twins conceived by infertility treatments (in vitro fertilization and ovulation induction) and those who were conceived spontaneously. Deliveries occurred between the years 1991 and 2021 in a tertiary medical center. A Kaplan–Meier survival curve was used to compare the cumulative incidence of childhood malignancies, and a Cox proportional hazards model was constructed to control for confounders. **Results**: During the study period, 11,986 twins met the inclusion criteria; 2910 (24.3%) were born following infertility treatments. No statistically significant differences were noted between the groups comparing the rate (per 1000) of childhood malignancies (2.0 in the infertility treatments group vs. 2.2 in the comparison group, OR 1.04, 95% CI 0.41–2.62; *p* = 0.93). Likewise, the cumulative incidence over time was comparable between the groups (log-rank test, *p* = 0.87). In a Cox regression model, controlling for maternal and gestational age, no significant differences in childhood malignancies were noted between the groups (adjusted HR = 0.82, 95% CI 0.49–1.39, *p* = 0.47). **Conclusions:** In our population, twins conceived following infertility treatments are not at an increased risk for childhood malignancies.

## 1. Introduction

In the last three decades the rate of twin births has been rising with infertility treatments as one of the major contributors to that increase [1]. Since first introduced, the use of infertility treatments, including in vitro fertilization (IVF) and ovulation induction (OI) has rose significantly [2,3,4]. For example, in 2019 it was estimated that over 8 million babies were born using IVF treatments and the annual numbers exceeded 500,000 births worldwide [2].

In singletons, infertility treatments are related to short term complications to the mother and child [5]. Maternal complications include pre-term birth (PTB) [6] gestational diabetes mellitus (GDM), preeclampsia, and premature rupture of membranes (PROM) [7]. Complications to the offspring include a higher risk for birth defects [8] and an increased risk for low birth weight (LBW) compared to spontaneous pregnancies [6]. Increased risk for short term complications, including preterm birth and low birth weight [9] and preeclampsia [10], was also found among twins conceived following infertility treatments. In addition to possible short-term implications, growing evidence suggests an association between infertility treatments and long-term morbidities in the offspring [11]. Previous studies show increased risk for elevated blood pressure, lower metabolic functioning [12], ophthalmic [13], infectious [14] and gastrointestinal morbidity [15].

The possible association between infertility treatments and long-term cancer risk has been studied with several underlying mechanisms suggesting this association. Epigenetic changes associated with infertility treatments or the underlying infertility [16] may lead to imprinting disorders, such as changes in gene expression and methylation levels, that increase the risk for future malignancy [17,18,19]. Infertility treatments may interrupt gene regulation and tumor suppression [20]. Thus, enhancing survival mechanisms that are not present normally in spontaneous pregnancies put these embryos at a higher risk for cancer [21]. Moreover, the administration of exogenous hormones may affect the fetus during crucial stages of differentiation and growth, consequently increasing the endocrine sensitivity for malignancy in the future [22,23].

Controversy exists in the literature with regard to the association between infertility treatments in singleton pregnancies and long-term risk for childhood malignancy. While some studies found an association [24,25,26], others show no increase in the overall risk [27,28]. These studies, however, focused on singletons and multiple gestations were excluded. Scarce data exist regarding fertility twins and long-term malignancies.

As the rates of the use of infertility treatments are rising, there is a growing interest regarding the possible long-term implication of infertility treatments in twins. Yet, with regard to childhood malignancy in twin gestations following infertility treatments, only limited data exist. A Nordic cohort study showed that in the infertility treatments group, children with malignancy were more often twins. However, the study also showed no overall higher risk among children born after infertility treatments compared to spontaneous conception [29].

In a cohort study conducted in the US, including data from five states, childhood cancer among twins and higher order multiples were examined. The data were taken after 1989 as infertility treatments became more frequent in later years. The results of this study were inconclusive while presenting a decreased risk for several types of cancer and suggesting an increased risk for several others. Moreover, higher order multiple births were not associated with childhood cancer. [30]. A Danish twin national cohort study compared the incidence of malignancies in twins following infertility treatments vs. those that were not. The results of this study found no cancer cases in the 3393 twins born after infertility treatments compared to nine cases in the 5130 singleton group. The follow-up period in this study was only 4.2 years, and the minimum observation time was only one year after delivery [31,32].

In this study, we performed a long-term follow-up of a relatively large number of offspring of twin pregnancies. We aimed to find whether an association exists between infertility treatments in this population and an increased risk for childhood malignancies compared to twins that were conceived spontaneously.

## 2. Methods

### 2.1. Ethical Approval

This study was conducted in accordance with the declaration of Helsinki; study protocol number 0357-19-SOR.

### 2.2. Study Population and Data Collection

A population based retrospective cohort analysis was conducted including all twins that were born between the years 1991 and 2021 at the Soroka University Medical Center (SUMC), the sole tertiary hospital in the southern region of Israel. SUMC served the entire population in this region and the single IVF unit in the region.

The independent variable was defined as mode of conception; twins conceived following infertility treatments (IVF and OI) vs. spontaneously conceived twins. Cases of perinatal death and twins with congenital malformations or chromosomal abnormalities were excluded from the analysis. The studied population included all forms of twins whether they were monozygotic or dizygotic.

The study combined two SUMC databases: the perinatal database from the Obstetric and Gynecologic department, as well as the children hospitalizations database.

The outcome variable was defined as the first pediatric hospitalization or encounter with the hospital of one of the twins with childhood malignancy diagnosed up to the age of 18. The incidence rates were per 1000 twins. All diagnoses were predefined in a set of International Classification of Diseases 9th edition (ICD 9) codes as documented in any hospitalization records. Malignant morbidity was based on hospitalization following a diagnosis of lymphoma, leukemia, brain, kidney, skin and others. A list of the grouped diagnoses and ICD 9 is presented in Table S2. A malignancy event was defined as the first hospitalization with any diagnoses from the malignancy codes list presented in Table S2. The follow-up time was from birth to an event or end of research period or at age 18.

### 2.3. Statistical Analysis

Univariable analysis was performed to compare dependent and background characteristics between the two study groups. Background and pregnancy characteristics included maternal age, gestational age in weeks, parity, smoking, obesity and preterm birth. The univariable analysis included Chi-square tests for categorical variables and *t*-tests or Man-Whitney U tests for continuous variables according to their distribution. All analyses were 2-sided. A two-sided α < 0.05 was defined as statistically significant.

Cumulative incidence rates of childhood malignancies were compared using Kaplan–Meier via the log-rank test to determine significant differences.

A Cox proportional survival hazard model was conducted to compare malignancy associated hospitalization risk among twins conceived following infertility treatments and those conceived spontaneously. The model adjusted for potential confounders based on the univariable analysis besides clinically important variables. The final model was chosen based on the best fit and minimal −2log likelihood.

## 3. Results

During the study period, 11,986 twins met the inclusion criteria; 2910 of whom (24.3%) were born following infertility treatments and 9076 (75.7%) were born spontaneously. In the infertility treatment group, 1908 twins were conceived by IVF treatments (15.9%) and 1002 twins were conceived by OI treatments (8.4%).

Table 1 presents maternal demographics and pregnancy characteristics of the study population. Mothers in the infertility treatment groups were significantly older as compared to the spontaneously conceived group. In the infertility treatments group, higher rates of preterm births were noted either before 37 weeks gestation (OR 1.51, 95% CI 1.39–1.65; *p* <0.001) or 34 weeks gestation (OR 1.36, 95% CI 1.22–1.55; *p* < 0.001). A lower parity in the infertility treatments group was noted as well.

During the study period, twenty-four offspring were diagnosed with malignancies (0.002% of the entire study population). Data regarding all children that were diagnosed with malignancy are presented in Supplementary Table S1. According to our data, in our study population, there was only one case where both twins had childhood malignancy. In addition, we had two children that had two different malignancies diagnosed during childhood.

Rates per 1000 twins by malignancy category are presented in Table 2. The rate of childhood malignancies between the groups showed no statistically significant differences, with 0.20% in the infertility treatments group compared to 0.20% in the spontaneously conceived group. Moreover, we have performed another analysis in which the studied population was defined as children that were diagnosed with childhood malignancies and have compared the rate of fertility treatments to the rest of the twin population. The results of this comparison show no increased rate of fertility treatments used in the group of children that were diagnosed with malignancy compared to the control group (25.0% vs. 24.3%; CI 0.41–2.62, *p* = 0.93).

**Figure 1:**
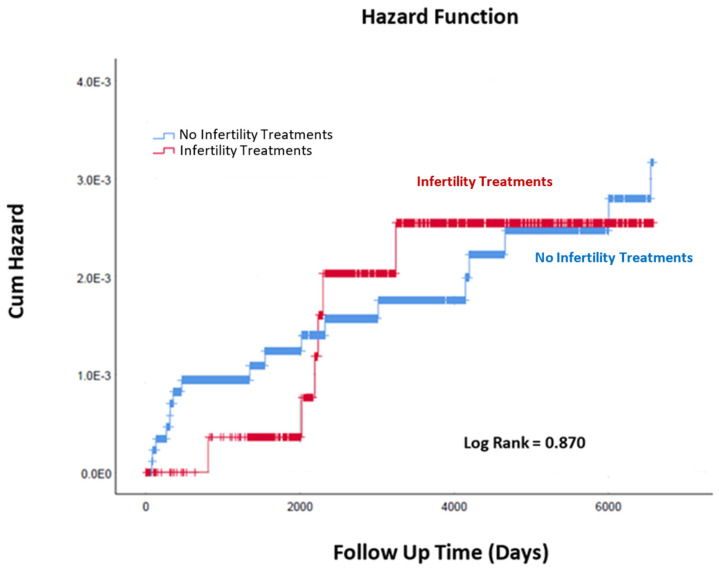
Cumulative incidence of childhood malignancies in twins born after infertility treatments and those who were not.

During the follow-up time of the study, the incidence of childhood malignancies was comparable between the groups over time (Kaplan–Meier log-rank, *p* = 0.87, Figure 1).

Table 3 presents the results of a Cox proportional hazards model for the risk of childhood malignancies between the two groups. The model adjusted for maternal and gestational age and showed no significant differences in risk for childhood malignancies between the groups (adjusted HR = 0.82, 95% CI 0.49–1.39, *p* = 0.47).

## 4. Discussion

In this population-based study with a long follow-up period, we found that twins conceived by infertility treatments are not at an increased risk for childhood malignancies compared with twins conceived spontaneously. These results are important given the high incidence of twins following infertility treatments.

In singleton pregnancies, several previous studies looked at the possible association between infertility treatments and long-term risk for childhood malignancies [25,26,27,28]. An earlier study by our group showed a statistically significant increased risk for childhood malignancies following infertility treatments [24]. This difference in the risk between singleton and twins may be the result of either a different uterine environment in twin pregnancies or a lack of statistical power in the current analysis.

Evidence found in the literature regarding this possible association in twin pregnancies is scarce [29,30,31,32].

Our study is in agreement with the results of those studies that showed no increased risk for malignancy in twins conceived following infertility treatments as compared to those who were conceived spontaneously.

With regard to other obstetric complications, it was previously shown that singletons who were conceived after infertility treatments are at an increased risk for low birth weight and preterm birth as compared to those who were conceived spontaneously [6]. Sunderam et al. have compared singletons conceived following infertility treatments to their twin counterparts; they found twins to be five times more likely for preterm delivery and six times more likely to be born with low birthweight [33].

A previous study among twins showed an increased risk for these same complications among infertility treatments twins as compared to their spontaneous counterparts [9].

Our results were in concordance with previous studies and show that infertility treatments twins are at a significantly higher risk for preterm birth compared to those who were not. Thus, the association of infertility treatments with adverse obstetric outcomes remains notable.

Our study shows noteworthy strengths, of which the main is that data collected for the study is based on two computerized databases presenting a large sample size. In addition, since our hospital is the only tertiary hospital in the southern region of Israel, the population is not selective. Therefore, representing a wide spectrum of patients of all socioeconomic backgrounds, thus reducing the likelihood of incorrect outcome data and bias. In addition, being the single tertiary center in the area that performs both the infertility treatments, the delivery and the pediatric follow-up provided us with a long follow-up time that was significantly longer compared to other studies. This enabled us to evaluate long-term childhood outcomes, including malignancies while controlling for other variables relating to the pregnancy and delivery.

There are limitations in our study that need to be addressed. Given the rarity of malignancy during childhood, our total number of malignancies was relatively small as compared to the studies conducted on singletons [24,34,35,36]. Much like previously published studies that looked at twins, the small number of cases we found limits our ability to look for possible correlations to each specific type of malignancy.

## 5. Conclusions

In conclusion, our research shows that twins conceived following infertility treatments are not at an increased risk for childhood malignancies. Given the increased use of infertility treatments globally, this information is of importance for physicians that are counseling parents regarding possible future implications of infertility treatments on offspring health. Further large-scale research is needed in order to further investigate any possible association between infertility treatments and twins’ childhood malignancy.

## Figures and Tables

**Table 1:** Maternal demographics and pregnancy characteristics at index pregnancy divided to twin pregnancy with and without a history of infertility treatments.

Characteristics		Infertility Treatments * N = 2910	No Infertility Treatments N = 9076	OR	95% CI	*p*-Value
Maternal Age (Years; Mean ± SD)		30.80 ± 5.33	29.36 ± 5.62			0.001
Gestational Age at delivery (Weeks; Mean ± SD)		35.07 ± 3.01	35.72 ± 2.93			0.152
Parity	1	56.5%	21.2%			0.001
	2–4	42.3%	50.4%			
	>5	1.20%	28.4%			
Smoking during pregnancy		0.7%	0.7%	1.11	0.67–1.83	0.68
Obesity during pregnancy		2.5%	1.6%	1.55	1.16–2.06	0.002
Preterm delivery	<37 weeks’ gestation	64.8%	54.9%	1.51	1.39–1.65	<0.001
	<34 weeks’ gestation	19.2%	14.8%	1.36	1.22–1.52	0.001>

* Infertility treatment group of the general study population: IVF—1908 patients (15.9%); OI—1002 patients (8.4%).

**Table 2:** Long-term malignancies (per 1000) of twins born to mothers treated and not treated by infertility treatments.

Childhood Malignancy	Infertility Treatments N = 2910 (per 1000)	No infertility Treatments N = 9076 (per 1000)	OR	95% CI	*p*-Value
Skin	1(0.3)	1(0.1)	3.12	0.19–49.89	0.4
Vagina vulva	1(0.3)	0	-	-	0.07
Testis	1(0.3)	0	-	-	0.07
Kidney	1(0.3)	4(0.4)	0.78	0.87–6.97	0.82
Ophthalmic	0	1(0.1)	-	-	0.57
Brain	1(0.3)	3(0.3)	1.04	0.11–9.99	0.97
Lymphoma	0	3(0.3)	-	-	0.32
Leukemia	1(0.3)	5(0.5)	0.62	0.07–5.34	0.66
Secondary	0	1(0.1)	-	-	0.57
Other	0	2(0.2)	-	-	0.42
Total	6(2.0)	18 (2.0)	1.04	0.41–2.62	0.93

**Table 3:** Cox multivariable regression model for the risk of childhood malignancies in twin children after infertility treatments vs. those that were spontaneously conceived controlling for other variables.

Variables	Adjusted HR	95% CI		*p*-Value
		Min	Max	
Infertility Treatments (Y/N)	0.826	0.491	1.389	0.472
Mother Age at Birth (years)	1.011	0.973	1.051	0.571
Gestational Age (Weeks)	0.876	0.818	0.939	<0.001

## Supplementary Materials

The following supporting information can be downloaded at: https://www.mdpi.com/article/10.3390/jcm12113728/s1, Table S1: Twenty-four cases of children that were diagnosed with a childhood malignancy during the study period. Table S2: Diagnosis Code.

## References

[1] Adashi E.Y. (2017). Seeing Double: A Nation of Twins from Sea to Shining Sea. Obstet. Anesth. Dig..

[2] Fauser B.C. (2019). Towards the global coverage of a unified registry of IVF outcomes. Reprod. Biomed. Online.

[3] Lindheim S.R., Glenn T.L., Smith M.C., Gagneux P. (2018). Ovulation Induction for the General Gynecologist. J. Obstet. Gynecol. India.

[4] Kamphuis E.I., Bhattacharya S., van der Veen F., Mol B.W.J., Templeton A. (2014). Are we overusing IVF?. BMJ.

[5] Chen M., Heilbronn L.K. (2017). The health outcomes of human offspring conceived by assisted reproductive technologies (ART). J. Dev. Orig. Health Dis..

[6] McDonald S.D., Han Z., Mulla S., Murphy K.E., Beyene J., Ohlsson A. (2009). Preterm birth and low birth weight among in vitro fertilization singletons: A systematic review and meta-analyses. Eur. J. Obst. Gynecol. Reprod. Biol..

[7] Silberstein T., Levy A., Harlev A., Saphier O., Sheiner E. (2014). Perinatal outcome of pregnancies following in vitro fertilization and ovulation induction. J. Mater.-Fetal Neonatal Med..

[8] Wen J., Jiang J., Ding C., Dai J., Liu Y., Xia Y., Liu J., Hu Z. (2012). Birth defects in children conceived by in vitro fertilization and intracytoplasmic sperm injection: A meta-analysis. Fertil. Steril..

[9] McDonald S.D., Han Z., Mulla S., Ohlsson A., Beyene J., Murphy K.E. (2010). Preterm birth and low birth weight among in vitro fertilization twins: A systematic review and meta-analyses. Eur. J. Obstet. Gynecol. Reprod. Biol..

[10] Okby R., Harlev A., Sacks K.N., Sergienko R., Sheiner E. (2018). Preeclampsia acts differently in in vitro fertilization versus spontaneous twins. Arch. Gynecol. Obstet..

[11] Hart R., Norman R.J. (2013). The longer-term health outcomes for children born as a result of IVF treatment: Part I—General health outcomes. Hum. Reprod. Updat..

[12] Sullivan-Pyke C.S., Senapati S., Mainigi M.A., Barnhart K.T. (2017). In Vitro fertilization and adverse obstetric and perinatal outcomes. Semin. Perinatol..

[13] Tsumi E., Lavy Y., Sheiner E., Barrett C., Harlev A., Hagbi Bal M., Wainstock T. (2021). Assisted reproductive technology and long-term ophthalmic morbidity of the offspring. J. Dev. Orig. Health Dis..

[14] Wainstock T., Sheiner E., Yoles I., Sergienko R., Landau D., Harlev A. (2019). Fertility treatments and offspring pediatric infectious morbidities: Results of a population-based cohort with a median follow-up of 10 years. Fertil. Steril..

[15] Shachor N., Wainstock T., Sheiner E., Harlev A. (2020). Fertility treatments and gastrointestinal morbidity of the offspring. Early Hum. Dev..

[16] Pinborg A., Loft A., Romundstad L.B., Wennerholm U.B., Söderström-Anttila V., Bergh C., Aittomäki K. (2016). Epigenetics and assisted reproductive technologies. Acta Obstet. Gynecol. Scand..

[17] Lazaraviciute G., Kauser M., Bhattacharya S., Haggarty P., Bhattacharya S. (2014). A systematic review and meta-analysis of DNA methylation levels and imprinting disorders in children conceived by IVF/ICSI compared with children conceived spontaneously. Hum. Reprod. Update.

[18] Tobi E.W., Goeman J.J., Monajemi R., Gu H., Putter H., Zhang Y., Slieker R.C., Stok A.P., Thijssen P.E., Müller F. (2014). DNA methylation signatures link prenatal famine exposure to growth and metabolism. Nat. Commun..

[19] Neelanjana M., Sabaratnam A. (2008). Malignant Conditions in Children Born After Assisted Reproductive Technology. Obstet. Gynecol. Surv..

[20] Lim D.H.K., Maher E.R. (2010). Genomic Imprinting Syndromes and Cancer. Adv. Genet..

[21] Källén B., Finnström O., Lindam A., Nilsson E., Nygren K.G., Olausson P.O. (2010). Cancer Risk in Children and Young Adults Conceived by In Vitro Fertilization. Pediatrics.

[22] Tournaire M., Devouche E., Espié M., Asselain B., Levadou A., Cabau A., Dunbavand A., Grosclaude P., Epelboin S. (2015). Cancer Risk in Women Exposed to Diethylstilbestrol in Utero. Therapies.

[23] Birnbaum L.S., Fenton S.E. (2003). Cancer and developmental exposure to endocrine disruptors. Environ. Health Perspect..

[24] Wainstock T., Walfisch A., Shoham-Vardi I., Segal I., Harlev A., Sergienko R., Landau D., Sheiner E. (2017). Fertility treatments and pediatric neoplasms of the offspring: Results of a population-based cohort with a median follow-up of 10 years. Am. J. Obstet. Gynecol..

[25] Weng S.S., Huang Y.T., Huang Y.T., Li Y.P., Chien L.Y. (2022). Assisted Reproductive Technology and Risk of Childhood Cancers. JAMA Netw. Open.

[26] Bal M.H., Harlev A., Sergienko R., Levitas E., Har-Vardi I., Zeadna A., Mark-Reich A., Becker H., Ben-David N., Naggan L. (2021). Possible association between in vitro fertilization technologies and offspring neoplasm. Fertil. Steril..

[27] Klip H., Burger C.W., de Kraker J., van Leeuwen F.E., on behalf of the OMEGA-project group (2001). Risk of cancer in the offspring of women who underwent ovarian stimulation for IVF. Hum. Reprod..

[28] Gilboa D., Koren G., Barer Y., Katz R., Rotem R., Lunenfeld E., Shalev V. (2019). Assisted reproductive technology and the risk of pediatric cancer: A population based study and a systematic review and meta analysis. Cancer Epidemiol..

[29] Sundh K.J., Henningsen A.K.A., Kallen K., Bergh C., Romundstad L.B., Gissler M., Pinborg A., Skjaerven R., Tiitinen A., Vassard D. (2014). Cancer in children and young adults born after assisted reproductive technology: A Nordic cohort study from the Committee of Nordic ART and Safety (CoNARTaS). Hum. Reprod..

[30] Puumala S.E., Carozza S.E., Chow E.J., Fox E.E., Horel S., Johnson K.J., McLaughlin C., Mueller B.A., Reynolds P., Von Behern J. (2009). Childhood Cancer among Twins and Higher Order Multiples. Cancer Epidemiol. Biomark. Prev..

[31] Pinborg A. (2005). IVF/ICSI twin pregnancies: Risks and prevention. Hum. Reprod. Update.

[32] Pinborg A., Loft A., Andersen A.N. (2004). Neonatal outcome in a Danish national cohort of 8602 children born after in vitro fertilization or intracytoplasmic sperm injection: The role of twin pregnancy: Neonatal outcome in IVF/ICSI twins and singletons. Acta Obstet. Gynecol. Scand..

[33] Sunderam S., Kissin D.M., Crawford S.B., Folger S.G., Jamieson D.J., Warner L., Barfield W.D. (2017). Assisted Reproductive Technology Surveillance—United States, 2014. MMWR Surveill. Summ..

[34] Spector L.G., Brown M.B., Wantman E., Letterie G.S., Toner J.P., Doody K., Ginsburg E., Williams M., Koch L., Schymura M. (2019). Association of In Vitro Fertilization with Childhood Cancer in the United States. JAMA Pediatr..

[35] Catford S.R., McLachlan R.I., O’Bryan M.K., Halliday J.L. (2017). Long-term follow-up of intra-cytoplasmic sperm injection-conceived offspring compared with in vitro fertilization-conceived offspring: A systematic review of health outcomes beyond the neonatal period. Andrology.

[36] Rudant J., Amigou A., Orsi L., Althaus T., Leverger G., Baruchel A., Bertrand Y., Nelken B., Plat G., Michel G. (2013). Fertility treatments, congenital malformations, fetal loss, and childhood acute leukemia: The ESCALE study (SFCE): Maternal and Birth Characteristics and AL. Pediatr. Blood Cancer.

