# Supplementary material for: Infertility Treatments Resulting in Twin Pregnancy: Does It Increase the Risk for Future Childhood Malignancy
Source: J Clin Med. 2023 May 29;12(11):3728. doi: 10.3390/jcm12113728 (PMC10253818; doi:10.3390/jcm12113728)
Supplement: Supplementary file 1 [file jcm-12-03728-s001.zip › jcm-2315748-supplementary.pdf]

Supplementary Table S1: Twenty-four cases of children that were diagnosed with a childhood malignancy during the study period.

Case number	Year of birth	Gender	Birthweight	Y/N Infertility Treatments	Follow up (years)	Cancer Type
**1**	1996	Male	2890	N	11.35	Leukemia
**2**	2002	Male	3040	N	0.96	Brain
**3**	1997	Male	2820	N	12.76	Lymphoma
**4**	2003	Female	2410	N	0.35	Kidney
**5**	1993	Female	2000	Y	8.88	Leukemia
**6**	2019	Male	1480	N	0.73	Kidney
**7**	2005	Male	2365	Y	5.53	Brain
**8**	2007	Female	2590	N	6.36	Kidney, Secondary
**9**	2007	Female	2630	N	8.25	Skin
**10**	2016	Female	2700	N	1.27	Brain
**11**	2020	Female	2840	N	0.87	Leukemia
**12**	2020	Female	2855	N	0.21	NOS
**13**	1997	Female	2455	Y	2.21	Vagina Vulva
**14**	2014	Female	1975	N	5.52	Kidney
**15**	1992	Male	2520	N	3.68	Leukemia
**16**	2004	Male	2850	N	16.44	Leukemia
**17**	2009	Female	2488	Y	6.29	Skin
**18**	2000	Male	2780	Y	6.00	Kidney
**19**	2011	Female	1227	N	0.23	Ophthalmic
**20**	1998	Male	2650	Y	6.12	Testis
**21**	2000	Female	1505	N	17.93	Lymphoma, Leukemia
**22**	1994	Male	3430	N	0.85	NOS
**23**	1996	Female	2995	N	11.49	Lymphoma
**24**	2015	Female	2095	N	4.23	Brain

Table S2.

Diagnosis Code	Diagnosis	Subgroup
193	MALIGNANT NEOPLASM OF THYROID GLAND	head and neck
1409	MALIGNANT NEOPLASM OF LIP, UNSP., VERMILION BORDER	head and neck
1420	MALIGNANT NEOPLASM OF PAROTID GLAND	head and neck
1450	MALIGNANT NEOPLASM OF CHEEK MUCOSA	head and neck
1479	MALIGNANT NEOPLASM OF NASOPHARYNX, UNSPECIFIED	head and neck
1950	MALIGNANT NEOPLASM OF HEAD, FACE, AND NECK	head and neck
1502	MALIGNANT NEOPLASM OF ABDOMINAL ESOPHAGUS	esophagus
1519	MALIGNANT NEOPLASM OF STOMACH, UNSPECIFIED	stomach

1540	MALIGNANT NEOPLASM OF RECTOSIGMOID JUNCTION	colon rectum
1541	MALIGNANT NEOPLASM OF RECTUM	colon rectum
V1006	PERSONAL HISTORY OF MAL.NEO.RECTUM,RECTOSIGMOID JUNCTION,ANUS	colon rectum
V1009	PERSONAL HISTORY MAL.NEOPL.OTHER SITES IN GASTROINTEST.TRACT	colon rectum
1550	MALIGNANT NEOPLASM OF LIVER, PRIMARY	liver
1570	MALIGNANT NEOPLASM OF HEAD OF PANCREAS	pancreas
1579	MALIGNANT NEOPLASM OF PANCREAS, PART UNSPECIFIED	pancreas
1580	MALIGNANT NEOPLASM OF RETROPERITONEUM	retroperitoneum
1589	MALIGNANT NEOPLASM OF PERITONEUM, UNSPECIFIED	peritoneum
1629	MALIGNANT NEOPLASM OF BRONCHUS AND LUNG, UNSPECIFIED	lung
2319	CARCINOMA IN SITU OF RESPIRATORY SYSTEM, PART UNSPECIFIED	lung
2391	NEOPLASM OF UNSPECIFIED NATURE OF RESPIRATORY SYSTEM	lung
V102	PERSONAL HISTORY MAL.NEOPL.OTHER RESPIR.AND INTRATHOR.ORGANS	lung
1642	MALIGNANT NEOPLASM OF ANTERIOR MEDIASTINUM	mediastinum
1649	MALIGNANT NEOPLASM OF MEDIASTINUM, PART UNSPECIFIED	mediastinum
1701	MALIGNANT NEOPLASM OF MANDIBLE	bone
1702	MAL.NEOPLASM OF VERTEBRAL COLUMN, EXCLUDING SACRUM & COCCYX	bone
1703	MALIGNANT NEOPLASM OF RIBS, STERNUM, AND CLAVICLE	bone
1704	MALIGNANT NEOPLASM OF SCAPULA AND LONG BONES OF UPPER LIMB	bone
1706	MALIGNANT NEOPLASM OF PELVIC BONES, SACRUM, AND COCCYX	bone
1707	MALIGNANT NEOPLASM OF LONG BONES OF LOWER LIMB	bone
1708	MALIGNANT NEOPLASM OF SHORT BONES OF LOWER LIMB	bone
1709	MAL.NEOPLASM OF BONE AND ARTICULAR CARTILAGE, SITE UNSPEC.	bone
V1081	PERSONAL HISTORY OF MALIGNANT NEOPLASM OF BONE	bone
1710	MAL.NEOPLASM OF CONNECTIVE AND SOFT TISSUE,HEAD,FACE,NECK	connective tissue
1712	MAL.NEOPLASM CONNEC.AND SOFT TISSUE,UPPER LIMB,INCL.SHOULDER	connective tissue
1713	MAL.NEOPLASM OF CONNECTIVE AND SOFT TISSUE LOWER LIMB,INCL.HIP	connective tissue
1714	MAL.NEOPLASM OF CONNECTIVE AND OTHER SOFT TISSUE OF THORAX	connective tissue
1715	MAL.NEOPLASM OF CONNECTIVE AND OTHER SOFT TISSUE OF ABDOMEN	connective tissue
1716	MAL.NEOPLASM OF CONNECTIVE AND OTHER SOFT TISSUE OF PELVIS	connective tissue
1717	MAL.NEOPLASM OF CONNECTIVE AND SOFT TISSUE OF TRUNK,UNSPECIFIED	connective tissue
1718	MAL.NEO.OF OTHER SPEC. SITES OF CONNECT.AND SOFT TISSUE	connective tissue
1719	MAL.NEOPLASM OF CONNECTIVE AND SOFT TISSUE,SITE UNSPECIFIED	connective tissue
173	OTHER MALIGNANT NEOPLASM OF SKIN	skin
1725	MALIGNANT MELANOMA OF SKIN OF TRUNK, EXCEPT SCROTUM	skin
1726	MALIGNANT MELANOMA OF SKIN OF UPPER LIMB, INCLUDING	skin

	SHOULDER	
1729	MELANOMA OF SKIN, SITE UNSPECIFIED	skin
1733	MALIGNANT NEOPLASM OF SKIN OF UNSPECIFIED PARTS OF FACE	skin
V1082	PERSONAL HISTORY OF MALIGNANT MELANOMA OF SKIN	skin
1741	MALIGNANT NEOPLASM OF CENTRAL PORTION OF FEMALE BREAST	breast
1830	MALIGNANT NEOPLASM OF OVARY	ovary
1840	MALIGNANT NEOPLASM OF VAGINA	vagina vulva
1844	MALIGNANT NEOPLASM OF VULVA, UNSPECIFIED	vagina vulva
2123	BENIGN NEOPLASM OF BRONCHUS AND LUNG	vagina vulva
2462	CYST OF THYROID	vagina vulva
20960	BENIGN CARCINOID TUMOR OF UNKNOWN PRIMARY SITE	vagina vulva
1869	MALIGNANT NEOPLASM OF OTHER AND UNSPECIFIED TESTIS	testis
V1047	PERSONAL HISTORY OF MALIGNANT NEOPLASM OF TESTIS	testis
1889	MALIGNANT NEOPLASM OF BLADDER, PART UNSPECIFIED	bladder
1890	MALIGNANT NEOPLASM OF KIDNEY, EXCEPT PELVIS	kidney
V1052	PERSONAL HISTORY OF MALIGNANT NEOPLASM OF KIDNEY	kidney
1905	MALIGNANT NEOPLASM OF RETINA	ophthalmic
V1084	PERSONAL HISTORY OF MALIGNANT NEOPLASM OF EYE	ophthalmic
1910	MALIGNANT NEOPLASM OF CEREBRUM, EXCEPT LOBES AND VENTRI-CLES	brain
1912	MALIGNANT NEOPLASM OF TEMPORAL LOBE	brain
1913	MALIGNANT NEOPLASM OF PARIETAL LOBE	brain
1914	MALIGNANT NEOPLASM OF OCCIPITAL LOBE	brain
1915	MALIGNANT NEOPLASM OF VENTRICLES	brain
1916	MALIGNANT NEOPLASM OF CEREBELLUM NOS	brain
1917	MALIGNANT NEOPLASM OF BRAIN STEM	brain
1918	MALIGNANT NEOPLASM OF OTHER PARTS OF BRAIN	brain
1919	MALIGNANT NEOPLASM OF BRAIN, UNSPECIFIED	brain
1920	MALIGNANT NEOPLASM OF CRANIAL NERVES	brain
1921	MALIGNANT NEOPLASM OF CEREBRAL MENINGES	brain
1928	MALIGNANT NEOPLASM OF OTHER SPECIFIED SITES OF NERVOUS SYS-TEM	brain
V1085	PERSONAL HISTORY OF MALIGNANT NEOPLASM OF BRAIN	brain
1940	MALIGNANT NEOPLASM OF ADRENAL GLAND	adrenal
1960	2ND AND UNSP.MAL.NEOPL.OF LYMPH NODES,HEAD,FACE AND NECK	secondary
1963	2ND AND UNSP.MAL.NEOPL.OF LYMPH NODES OF AXILLA+UPPER LIMB	secondary
1970	SECONDARY MALIGNANT NEOPLASM OF LUNG	secondary
1980	SECONDARY MALIGNANT NEOPLASM OF KIDNEY	secondary
1983	SECONDARY MALIGNANT NEOPLASM OF BRAIN AND SPINAL CORD	secondary
1985	SECONDARY MALIGNANT NEOPLASM OF BONE AND BONE MARROW	secondary
19889	SECONDARY MALIGNANT NEOPLASM OF OTHER SPECIFIED SITES	secondary
202	OTHER MALIGNANT NEOPLASMS OF LYMPHOID & HISTIOCYTIC TISSUE	lymphoma
2001	LYMPHOSARCOMA	lymphoma
2002	BURKITT'S TUMOR OR LYMPHOMA	lymphoma
2021	MYCOSIS FUNGOIDES	lymphoma
2028	OTHER MALIGNANT LYMPHOMAS	lymphoma
20011	LYMPHOSARCOMA INVOLVING LYMPH NODES OF HEAD, FACE, AND	lymphoma

	NECK	
20012	LYMPHOSARCOMA INVOLVING INTRATHORACIC LYMPH NODES	lymphoma
20013	LYMPHOSARCOMA INVOLVING INTRA-ABDOMINAL LYMPH NODES	lymphoma
20020	BURKITT'S TUMOR OR LYMPHOMA, UNSP.SITE, EXTRANODAL & SOLID ORGAN SITES	lymphoma
20021	BURKITT'S TUMOR;LYMPHOMA INVOLV.LYMPH NODES HEAD,FACE,NECK	lymphoma
20023	BURKITT'S TUMOR,LYMPHOMA INVOLV.INTRA-ABDOMINAL LYMPH NODES	lymphoma
20025	BURKITT'S TUMOR;LYMPHOMA INV.LYMPH NODES IN-GUIN.REG./LOWER LIMB	lymphoma
20028	BURKITT'S TUMOR;LYMPHOMA INVOLVING LYMPH NODES MULTIPLE SITES	lymphoma
20151	HODGKIN'S DIS.NODULAR SCLEROSIS,INVOLVING HEAD,FACE,NECK	lymphoma
20152	HODGKIN'S DIS.NODULAR SCLEROSIS,INVOLV.INTRATHORACIC LYMPH N.	lymphoma
20161	HODGKIN'S DIS.MIXED CELLULARITY,INVOLVOLVING HEAD,FACE,NECK	lymphoma
20162	HODGKIN'S DIS.MIXED CELLULARITY,INVOLV.INTRATORACIC LYMPH N.	lymphoma
20190	HODGKIN'S DIS.,UNSP. TYPE, UNSP. SITE, EXTRANODAL & SOLID OR-GAN SITES	lymphoma
20191	HODGKIN'S DIS.UNSPECIFIED TYPE,INVOLVING HEAD,FACE,NECK	lymphoma
20192	HODGKIN'S DISEASE UNSP.TYPE,INVOLV.INTRATHORACIC LYMPH NODES	lymphoma
20193	HODGKIN'S DISEASE UNSP.TYPE,INVOLV.INTRA-ABDOMINAL LYMPH NODES	lymphoma
20196	HODGKIN'S DIS.UNSPECIFIED TYPE,INVOLV.INTRAPELVIC LYMPH NODES	lymphoma
20198	HODGKIN'S DIS.UNSPECIFIED TYPE,INVOLVING MULTIPLE SITES	lymphoma
20200	NODULAR LYMPHOMA, UNSP.SITE, EXTRANODAL & SOLID ORGAN SITES	lymphoma
20202	NODULAR LYMPHOMA INVOLVING INTRATHORACIC LYMPH NODES	lymphoma
20210	MYCOSIS FUNGOIDES, UNSP.SITE, EXTRANODL AND SOLID ORGAN SITES	lymphoma
20211	MYCOSIS FUNGOIDES INVOLVING LYMPH NODES OF HEAD, FACE, AND NECK	lymphoma
20212	MYCOSIS FUNGOIDES INVOLVING INTRATHORACIC LYMPH NODES	lymphoma
20213	MYCOSIS FUNGOIDES INVOLVING INTRA-ABDOMINAL LYMPH NODES	lymphoma
20280	OTHER MALIGNANT LYMPHOMAS, UNSP.SITE, EXTRANODAL & SOLID ORGAN SITES	lymphoma
20281	OTHER MALIGNANT LYMPHOMAS INVOLV.LYMPH NODES HEAD,FACE,NECK	lymphoma
20282	OTHER MALIGNANT LYMPHOMAS INVOLVING INTRATHORACIC LYMPH NODES	lymphoma
20283	OTHER MALIGNANT LYMPHOMAS INVOLVING INTRA-ABDOMINAL LYMPH NODES	lymphoma
20285	OTHER MALIGNANT LYMPHOMAS INVOLVING INGUINAL REG./LOWER LIMB	lymphoma
20293	UNSP.MAL.NEO.OF LYMPHOID/HISTIOCYTIC,INTRA-ABDOMINAL LYMPH N.	lymphoma
V1071	PERSONAL HISTORY OF LYMPHOSARCOMA AND RETICULOSARCOMA	lymphoma
V1072	PERSONAL HISTORY OF HODGKIN'S DISEASE	lymphoma

V1079	PERSONAL HISTORY OF OTHER LYMPHATIC + HEMATOPOIETIC NEOPL.	lymphoma
204	LYMPHOID LEUKEMIA	leukemia
2040	LYMPHOID LEUKEMIA, ACUTE	leukemia
2040	LYMPHOID LEUKEMIA, ACUTE__ 1992 פרט	leukemia
2080	LEUKEMIA OF UNSPECIFIED CELL TYPE, ACUTE__1992 פרט	leukemia
2089	UNSPECIFIED LEUKEMIA___1992 פרט	leukemia
20300	MULTIPLE MYELOMA WITHOUT MENTION OF REMISSION	leukemia
20400	ACUTE LYMPHOID LEUKEMIA WITHOUT MENT.OF HAVING ACHIEVED REMISSION	leukemia
20400	LYMPHOID LEUKEMIA, AC., WITHOUT MENTION OF REMISSION	leukemia
20401	LYMPHOID LEUKEMIA, ACUTE, IN REMISSION	leukemia
20402	ACUTE LYMPHOID LEUKEMIA IN RELAPSE	leukemia
20500	MYELOID LEUKEMIA, AC., WITHOUT MENTION OF HAVING ACHIEVED REMISS.	leukemia
20500	MYELOID LEUKEMIA, AC., WITHOUT MENTION OF REMISSION	leukemia
20502	ACUTE MYELOID LEUKEMIA IN RELAPSE	leukemia
20510	MYELOID LEUKEMIA, CHR., WITHOUT MENTION OF HAVING ACHIEVED REMIS.	leukemia
20530	MYELOID SARCOMA WITHOUT MENTION OF REMISSION	leukemia
20720	MEGAKARYOCYTIC LEUKEMIA WITHOUT MENTION OF REMISSION	leukemia
20800	AC.LEUKEMIA OF UNSP.CELL TYPE WITHO.MENT.OF HAVING ACHIEVED REMI.	leukemia
20800	LEUKEMIA, UNSP. CELL TYPE, AC., WITHOUT MENTION OF REMISSION	leukemia
20801	LEUKEMIA OF UNSP. CELL TYPE, AC., IN REMISSION	leukemia
20890	UNSP. LEUKEMIA WITHOUT MENTION OF HAVING ACHIEVED REMIS-SION	leukemia
20890	UNSP. LEUKEMIA WITHOUT REMISSION	leukemia
27788	TUMOR LYSIS SYNDROME	leukemia
28522	ANEMIA IN NEOPLASTIC DISEASE	leukemia
V1061	PERSONAL HISTORY OF LYMPHOID LEUKEMIA	leukemia
V1062	PERSONAL HISTORY OF MYELOID LEUKEMIA	leukemia
V1069	PERSONAL HISTORY OF OTHER LEUKEMIA	leukemia
1735	OTHER MALIGNANT NEOPLASM OF SKIN OF TRUNK, EXCEPT SCROTUM	OTHER
1952	MALIGNANT NEOPLASM OF ABDOMEN	OTHER
2390	NEOPLASM OF UNSPECIFIED NATURE OF DIGESTIVE SYSTEM	OTHER
2392	NEOPLASM OF UNSPECIFIED NATURE OF BONE, SOFT TISSUE, AND SKIN	OTHER
2395	NEOPLASM OF UNSPECIFIED NATURE OF OTHER GENITOURINARY OR-GANS	OTHER
2396	NEOPLASM OF UNSPECIFIED NATURE OF BRAIN	OTHER
2398	NEOPLASM OF UNSPECIFIED NATURE OF OTHER SPECIFIED SITES	OTHER
20002	RETICULOSARCOMA INVOLVING INTRATHORACIC LYMPH NODES	OTHER
23989	NEOPLASM OF UNSPECIFIED NATURE OF OTHER SPECIFIED SITES	OTHER
